# Hospital Meetings, &c.

**Published:** 1899-05-27

**Authors:** 


### HOSPITAL MEETINGS, &C.

### ADDENBROOKE'S HOSPITAL.

At the adjourned quarterly court of the Addenbrooke's Hospital, Cambridge, the question of the administration was brought forward again. Mr. Adeane moved the following resolution:—

"That a committee consisting of twelve governors of the hospital, four from the county, four from the borough, and four from the University, be appointed to consider the administration and financial position of the hospital; and especially to consider the desirability of forming an advisory council, representative as far as possible of the county, the borough, and the University, to make recommendations to the general courts on all questions of the administration, finance, and general policy of the hospital. The committee to have power to call in members of the honorary medical staff and other officers of the hospital as assessors."

This motion was passed unanimously, and Mr. Adeane then proceeded to propose that in view of the unsatisfactory result of the present administration and the falling off in the funds of the hospital, the advisory council referred to in the motion should consist of a limited number of members representing all interests, and to meet the special circumstances of their statutes that it should have no executive power. Dr. Waraker vehemently opposed this proposal of Mr. Adeane's, and in effect stated that the present administration and the system pursued were all that could be desired, and that no change could produce better results. He said that subscriptions had touched the bottom, but he did not give his reasons for thinking this. He said there was no hope of increased subscriptions, and the only course that commended itself to him was to let things remain as they were, and see what happened. Some discussion followed Dr. Waraker's non-possumus speech, and votes were taken on the motion, with the result that thirty-three voted for it, and thirteen against the proposition, which was, therefore, carried. We publish a small note on the subject from the Cambridge *Daily News*:—

"The Administration of Addenbrooke's Hospital.

"Subscriptions to the hospital will not go up until peace is restored, said Dr. Waraker, in effect, at the meeting of the governors of Addenbrooke's Hospital on Friday. Dr. Waraker might have added, although he did not do so, that peace will not be restored until the causes of dispeace are removed. If there is one thing that is perfectly clear in connection with Addenbrooke's Hospital, it is that the hospital is crippled by an antiquated system of management. This is a system which in appearance confers upon all subscribers of two guineas a vote in the management of the hospital; but in reality it does nothing of the kind. In theory all governors have a right to attend the weekly board; in fact, the only governors who take part in the work of the weekly board are a few who happen to live in Cambridge and who have leisure. The great body of the governors are, in so far as the administrative work of the weekly board is concerned, disfranchised. They cannot attend themselves; they are not allowed to choose representatives of their own way of thinking to attend on their behalf. In hospital finance it has been found that a voice in the management either directly or through representatives, is a genuine inducement to persons to become governors. This inducement Addenbrooke's Hospital cannot offer, save to a few leisured persons who live in Cambridge. Dr. Waraker deprecated the idea of the management falling into the hands of an oligarchy. An oligarchy which is representative is not an oligarchy at all. Save for the select governors, who may be swamped at any time, the weekly board of Addenbrooke's Hospital, as at present constituted, is an oligarchy entirely self elected. The strongest argument for the creation of an advisory committee is that it will introduce the representative element in as far as it seems possible under the present constitution to do so. It will give a voice to the county subscribers. Moreover, it may help to bring about that change in the system of administration which all dispassionate observers believe to be essential to the prosperity of the hospital. The creation of an advisory committee cannot be regarded in any sense as final, but it is a step in the right direction, and is on that account to be approved."

### EAST LONDON HOSPITAL FOR CHILDREN.

The festival dinner of the East London Hospital for Children and Dispensary for Women, Shadwell, E., was held on Monday evening, 15th inst., at the Hotel Metropole, under the presidency of His Royal Highness the Duke of Connaught. Among those present were the Earl of Errol, Mr. Spencer Charrington, M.P., Lord Egerton of Tatton, Lord Huntingfield, the Bishop of London, Colonel and Sheriff Probyn, Lord de Manley, the Bishop of Bristol, Lord Napier of Magdala, Sir William Corry, Lord Loch, Major J. Roper Parkington, Mr. H. Griffith, Mr. H. W. Trinder (chairman of the Board of Management), and Mr. Thomas Hayes (secretary).

The toast of "The Queen" having been duly honoured,

The Chairman, in giving "The Prince and the Princess of Wales and the rest of the Royal Family," said that all present knew the great interest the Prince of Wales especially had taken in hospitals, and the success which had attended his appeal for funds for those great institutions. They all felt grateful to him for what he had done. This hospital alone had received £250 from the Prince's fund. (Applause.)

The toast was enthusiastically honoured.

The Chairman, upon rising to propose "Success to the East London Hospital for Children," was received with loud cheers. This, he said, was their most important toast. In giving it he might have to weary those present with a few statistics. A very excellent report had been drawn up giving particulars of the history and work carried out by the hospital since 1868, when it was commenced in a very small way by Dr. and Mrs. Heckford. (Applause.) He believed the memory of that gentleman was very much venerated by all who took an interest in the hospital. (Hear, hear.) At first two houses were taken, in which ten beds were provided. Within a year the necessity for the hospital was fully proved by the increase in the number of beds to 30. From that time the accommodation had gradually increased, and there were now 93 beds in the general wards, and 10 in the isolation blocks for infectious cases. The hospital was very much indebted to one who, alas! was taken from them last year—the Earl of Strafford, who was for 28 years the president of the hospital. It was also indebted to many members of the aristocracy who had taken an interest in it, and had laid foundation stones and assisted the hospital under different circumstances. He was glad to say that his sister-in-law, the Duchess of Albany, was the last to open the hospital in 1896, after it had been closed for several months, owing to the carrying out of very necessary sanitary arrangements. The hospital was situated in a locality which was unknown to a great many people—he regretted to say it was unknown to himself, but he believed its situation was admirably adapted to meet the great want which existed of a hospital in the eastern part of London, and to treat the very large number of cases that came to it. Of course it laboured under a great disadvantage when compared with the Great Ormond Street Hospital—to which it was only second in size and importance—it being less known to the wealthier people than that great institution. He wished to impress upon those present the claims of the East London Hospital for Children, and he was pleased to see many ladies present, for what lady was there who did not take the warmest interest in all hospitals, and especially those for children? (Hear, hear.) It was satisfactory to find that since the foundation of the hospital no fewer than 26,236 in-patients and 476,284 out-patients had received attention there. (Applause.) He mentioned these numbers because numbers proved a great deal. It must be a satisfaction to all who were taking part in that festival dinner to know how great had been the work carried out by the hospital. (Hear, hear.) It was terrible to think that but for its existence the large number of people to whom he had referred would have had no alleviation to their sufferings. The great event of last year had been the opening of the Convalescent Home, so kindly named after the late Duchess of Teck, the "Princess Mary" Convalescent Home, at Bognor. It was, he believed, a charming building. It was constructed for 28 beds, but he regretted to learn that at present, owing to the want of funds, only 20 of the beds were available. The home was only opened towards the end of last year, but before the year was out 40 children had been treated there. The home cost £5,800, and he was happy to say that the whole of this had been paid —not only for the building, but for the freehold site on which it stood. There was, however, still required for the furnishing and equipment of the hospital about £1,400. This hospital, not unlike others, was incessantly advancing with the times. The medical authorities—he had no doubt they were perfectly right—were rather exigeant in the present day, and insisted upon having everything up to the latest principles of medical knowledge. It had been deemed necessary to provide a new operating theatre, and he believed this was already far advanced. Everybody knew that a modern hospital was considered quite imperfect without such a building. This, however, meant money, and he believed it would involve the expenditure of £1,000 or more. Towards this the Prince of Wales's Hospital Fund had, as he had stated, already given £250. (Applause.) From the Hospital Sunday and Saturday Funds money had also been received, but more was required, and he appealed most warmly—and he hoped he would not do so in vain—to the public who supported great institutions such as this in the hope that they would come forward and help the East London Hospital. (Applause.) The new home at Bognor would, he believed, require £600 a year to keep it going, and that excellent and useful building having been constructed, it would be a great misfortune if the full number of beds could not be used. The appeal made last year on behalf of the hospital brought in some £1,800, and he should very much regret if the present meeting were not even more successful. (Applause.)

The toast was drunk with enthusiasm.

Mr. H. W. Trinder, in reply, said there were two matters which they wanted to keep before their supporters: The first was the wants of the hospital, and the second the wants of the convalescent home. The two formed one institution, but to a great extent they were distinct. Having invited those present to pay either the hospital or the home at Bognor a visit, the speaker referred to the beneficial results of the work of the institutions, and speaking particularly of the hospital, said that many of the children who went there were half-starved, and there were some whose only illness was starvation. He then emphasised the several points already alluded to by His Royal Highness with regard to the financial needs of the hospital, but said the new operating theatre would cost about £1,500, of which they had at present only £250. They also required a new laundry, but for that they had had to wait until they could secure some additional land. This, however, had now been secured. Other wants included additional rooms for their nurses and servants, and a separate ward for the treatment of whooping-cough cases. Altogether, if they had the money, they could at once dispose of £5,000 in these various directions. Mr. Trinder paid a warm tribute, not only to the ability of the hon. medical officer of the convalescent home, but also to the great personal interest he took in the patients who were sent there. He added that at Bognor they wanted to provide an isolation ward and a laundry, and to so lay out their grounds that the children might get the benefit of the sea air without having necessarily to go down to the beach. (Applause.)

The Bishop of London gave "The Board of Management" in a few appropriate sentences, and Viscount Falkland (the vice-chairman) briefly returned thanks.

"The Medical and Resident Staff" was submitted by the Earl of Erroll, who eulogised the services of the several members of the staff, and whose kind words were acknowledged by Dr. A. M. Gossage.

The Secretary then, amid applause, announced donations amounting to £2,255 10s., including one from the chairman of £25.

The toast of "The Visitors and the Ladies," proposed by Major Parkington, was responded to by Colonel and Sheriff Probyn.

Vocal and instrumental music enlivened the proceedings.

### NORTH-EASTERN HOSPITAL FOR CHILDREN.

The thirty-first annual meeting of the governors of the North-Eastern Hospital for Children was held at the hospital on Monday, the 15th inst., Mr. Walter Johnson presiding. The annual report for 1898 showed that during the year the in-patients numbered 764, as compared with 772 in 1897; the out-patients 15,554, as against 14,947 in the preceding year; and the attendances were 58,551, an increase of 4,199 on the total number stated in the last annual report. The expenditure for the year amounted to £6,091, and the receipts were £6,137, of which £1,563 was received from annual subscriptions, £2,428 from donations, £569 from the Prince of Wales's Hospital Fund and the Hospital Saturday and Hospital Sunday Funds, and the remainder from offertories, collecting boxes, patients' payments, convalescent homes, and invested property. In addition, the sum of £1,750 from legacies was placed to the building fund account. A complete Röntgen Rays apparatus, which had been of great benefit to the hospital, was presented by the Duke of Newcastle. In consideration of the possibility of unfair advantage being taken of the privileges of the hospital by the well-to-do, inquiries had been instigated into the circumstances of the parents of out-patients. Out of 89 cases in which information was sought, 45 were found unsuitable, and refused further treatment. Some alterations had been made in the distribution of the duties of the medical officers resident at the hospital. The senior resident will henceforth be known as "resident medical officer." The generosity of all friends and patrons of the hospital was appealed to on behalf of the new buildings which were to be added to the hospital. A piece of land adjoining the present buildings had been purchased for £4,940, and it was confidently hoped that the building would be commenced some time this year. The pressing need for extended accommodation was strongly commented on in the official report of a visiting committee of the Prince of Wales's Hospital Fund. It was there stated that in examining the hospital the visitors were chiefly impressed by the limited accommodation, and the excessively crowded state of every department, especially the out-patients'.

The Chairman, in moving the adoption of the report, referred with deep regret to the deaths of Mr. Joseph Gurney Barclay, who had been president of the hospital since its foundation in 1867, and of Alderman Sir Stuart Knill, Bart., for seven years an influential and valued member of the committee. Reference was made to the useful work done by the recently-established dental department, and to the improvement in the hospital by the introduction of the electric light. The valuable aid given to the hospital funds by the various local societies was gratefully mentioned.

The Rev. W. G. Morcom, in seconding the resolution, emphasised the need for the enlargement of the hospital buildings. No isolation ward for infectious cases exists at present, and, for want of it, every year numbers of children have to be turned away.

A resolution for the re-election of the officers and committee was unanimously carried. The Chairman, in referring to the vacant presidentship, stated that a correspondence with regard to that matter had been opened with Lord Amherst of Hackney. The committee greatly desire to have for president one of our Royal princes, but nothing is as yet definitely arranged concerning the election.

A vote of thanks was passed to the medical and nursing staffs, and to the secretary, Mr. Glenton-Kerr, for their services during the year.

